# Supplementary material for: Controlling an effector with eye movements: The effect of entangled sensory and motor responsibilities
Source: PLoS One. 2022 Feb 3;17(2):e0263440. doi: 10.1371/journal.pone.0263440 (PMC8812848; doi:10.1371/journal.pone.0263440)
Supplement: Attachment — Submitted filename: Response to Reviewers.pdf [file pone.0263440.s003.pdf]

Response to Reviewers

We again want to thank the reviewers for their thoughtful responses. In this letter, we have included the initial reviewer comments as well as our responses. The initial comments are numbered and our responses are shown in bold below each numbered comment. The line numbers correspond to the changes in "Revised Manuscript with Track Changes". We also made 2 other minor changes to further clarify details in the manuscript.

Reviewer 1:

1. Most of my comments have been adequately addressed by the authors.
   Regarding the equivalence test replacing the t-test, I miss the justification for the equivalence limits chosen. 1 cm might be a reasonable choice, but especially if the equivalence bounds are not set before the initial data analysis, the rationale behind the choice should be briefly explained in the manuscript.

This is a good suggestion. We added a brief explanation regarding the choice of 1cm for the equivalence interval. To measure the hand motion, we placed a motion capture marker on a stylus, which was attached to the participant's finger. During experimental conditions including hand motion, the participants were instructed to touch the tip of the stylus to the center of the target. The tip of the stylus had a diameter of 1cm. Therefore, if the eye fixation error was within 1cm of the hand error, we considered this to be equivalent, as reflected by the 1cm equivalence interval. We addressed this response in the manuscript (p. 9, ll. 332 - 335).

2. P 11, ll. 436-437: typo: "despite" is repeated

Good catch. This has been fixed (p. 10, ll. 396).
